# Structural Brain Alterations Associated with Rapid Eye Movement Sleep Behavior Disorder in Parkinson’s Disease

**DOI:** 10.1038/srep26782

**Published:** 2016-06-01

**Authors:** Soufiane Boucetta, Ali Salimi, Mahsa Dadar, Barbara E. Jones, D. Louis Collins, Thien Thanh Dang-Vu

**Affiliations:** 1 Center for Studies in Behavioural Neurobiology, PERFORM Center and Dpt of Exercise Science, Concordia University, 7141 Sherbrooke St. West, Montréal, Québec, H4B 1R6 Canada; 2 Centre de Recherche de l’Institut Universitaire de Gériatrie de Montréal and Dpt of Neurosciences, Université de Montréal, 4545 Chemin Queen Mary, Montréal, Québec, H3W 1W4 Canada; 3 Montreal Neurological Institute, McGill University, 3801 University Street, Montréal, Québec, H3A 2B4 Canada

## Abstract

Characterized by dream-enactment motor manifestations arising from rapid eye movement (REM) sleep, REM sleep behavior disorder (RBD) is frequently encountered in Parkinson’s disease (PD). Yet the specific neurostructural changes associated with RBD in PD patients remain to be revealed by neuroimaging. Here we identified such neurostructural alterations by comparing large samples of magnetic resonance imaging (MRI) scans in 69 PD patients with probable RBD, 240 patients without RBD and 138 healthy controls, using deformation-based morphometry (p < 0.05 corrected for multiple comparisons). All data were extracted from the Parkinson’s Progression Markers Initiative. PD patients with probable RBD showed smaller volumes than patients without RBD and than healthy controls in the pontomesencephalic tegmentum, medullary reticular formation, hypothalamus, thalamus, putamen, amygdala and anterior cingulate cortex. These results demonstrate that RBD is associated with a prominent loss of volume in the pontomesencephalic tegmentum, where cholinergic, GABAergic and glutamatergic neurons are located and implicated in the promotion of REM sleep and muscle atonia. It is additionally associated with more widespread atrophy in other subcortical and cortical regions whose loss also likely contributes to the altered regulation of sleep-wake states and motor activity underlying RBD in PD patients.

RBD is characterized by episodes of dream-enactment behaviors, during which abnormal motor activity during REM sleep often results in dramatic movements such as punching, flailing or jumping out of the bed^1^. It has been suggested that RBD results from an alteration of brainstem neural systems controlling motor inhibition during REM sleep^2^,^3^. Lesions in the brainstem, particularly involving the mesencephalic, pontine or medullary reticular formation (RF), were originally shown in animal models to produce REM sleep without atonia and acting out dreams^4^,^5^,^6^,^7^. In humans, neuropathological studies of small series of RBD patients have revealed loss of neurons in the region of the pontomesencephalic tegmentum (PMT)^8^. To generalize these findings, neuroimaging studies have compared regional brain volumes between subjects with idiopathic RBD (i.e., in the absence of any other neurological disorder) and subjects without. Studies of 12 to 26 idiopathic RBD patients demonstrated subtle structural alterations (indexed by MRI diffusion parameters) in regions compatible with the PMT and RF^9^,^10^, and the presence of altered regional brain volumes in these areas was reported in one study^11^ but not in two others^9^,^12^, which questions the consistency of pontine neuronal loss in idiopathic RBD.

Beyond these studies of ‘idiopathic’ RBD, recent studies have focused on RBD in presence of neurodegenerative disorders. Indeed, there is increasing evidence that RBD is associated with α-synucleinopathies such as PD, dementia with Lewy bodies and multiple system atrophy. The prevalence of RBD in the general population is approximately 0.5%,^13^ while this prevalence increases noticeably in PD (37 to 47%)^14^,^15^,^16^. In fact, RBD is considered as an early symptom of α-synucleinopathies^17^. Yet not all patients with these syndromes are affected by RBD. Besides the occurrence of dream-enactment behaviours, PD patients with RBD show differences in their clinical characteristics compared to those without, such as more severe cognitive and motor impairments, higher sleepiness levels and more frequent hallucinations^18^. It remains unclear whether these differences are subtended by specific brain anatomical changes. Indeed, few neuroimaging studies have investigated alterations associated with RBD in PD patients. An MRI study of 24 PD patients with RBD compared to 12 patients without showed subtle structural alterations in the PMT reflected by a decrease of neuromelanin-sensitive signal in the locus coeruleus/subcoeruleus within the PMT^19^. However, no significant change in regional brain volume was observed between the two PD groups in that study when using voxel-based morphometry (VBM).

Therefore evidence in RBD patients for extensive neurostructural alterations encompassing systems regulating REM sleep atonia remains to be clearly established. Most neuroimaging studies of RBD used a video-polysomnography assessment to provide a comprehensive diagnostic confirmation of RBD. This approach, while favoring a high internal validity, usually precludes the inclusion of large samples of participants. Yet resorting to larger cohorts of RBD patients – screened with validated questionnaires – might constitute a useful approach to demonstrate consistent patterns of structural brain changes associated with this condition. In the present study, we characterized brain abnormalities associated with the presence of RBD in a large population of PD patients. We used deformation-based morphometry (DBM) analyses on T1-weighted MRI images to identify significant differences in regional brain volumes between PD patients with probable RBD (pRBD) and PD patients without RBD (noRBD), based on the RBD screening questionnaire (RBDSQ)^20^. DBM allows a detection of volume differences in both grey and white matter, and was found more sensitive than conventional VBM methods in detecting volume changes in early-stage PD patients^21^. All data were extracted from the database of the Parkinson’s Progression Markers Initiative (PPMI), a multicenter program aimed at identifying progression biomarkers in newly diagnosed PD patients^22^.

## Results

Clinical and imaging data of 69 pRBD, 240 noRBD and 138 healthy controls (HC) were obtained from the PPMI. Clinical data of the three groups were compared and are illustrated in table 1. There was no significant difference in demographic characteristics (age, sex and level of education) between the three groups. However, in the case of family history of PD, the two patient groups had a higher incidence of PD in their families compared to controls. Also, per the inclusion criteria, the two patient groups were all newly diagnosed with PD (≤2 years) and did not significantly differ in time since PD diagnosis. For PD-related symptoms, pRBD showed higher rating scales in Movement Disorder Society-Unified Parkinson’s Disease Rating Scale (MDS-UPDRS) I and II than noRBD. They also showed more severe daytime sleepiness using Epworth Sleepiness Scale (ESS), olfactory deficit using University of Pennsylvania Smell Identification Test (UPSIT), depression and anxiety scores. Finally, pRBD showed lower cognitive performance than noRBD at the Symbol Digit Modalities Test (SDMT), Semantic Verbal Fluency Test (SVFT) and Benton’s Judgment of Line Orientation (BJLO), as well as more pronounced autonomic dysfunction.

**Figure 1:**
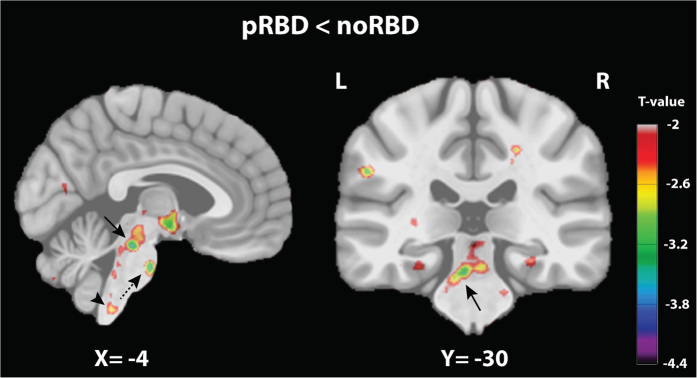
Volume change comparison between PD patients with (pRBD) and without (noRBD) probable RBD in the brainstem. Thresholded *t* maps (at *P* < 0.05 corrected for multiple comparisons) superimposed onto Montreal Neurological Institute 152 brain imaging template (i.e., a normalized average of 152 3D T1-weighted MRI scans from a normative adult population). The color-coded bar represents t values and the display is thresholded at *P* < 0.05. Left, sagittal plane showing smaller volume in the pontomesencephalic tegmentum (PMT) (arrow), the ventral medulla (arrow head), and base of pons (dashed arrow). Right, coronal plane showing the bilateral smaller volume in the PMT area (arrow).

**Figure 2:**
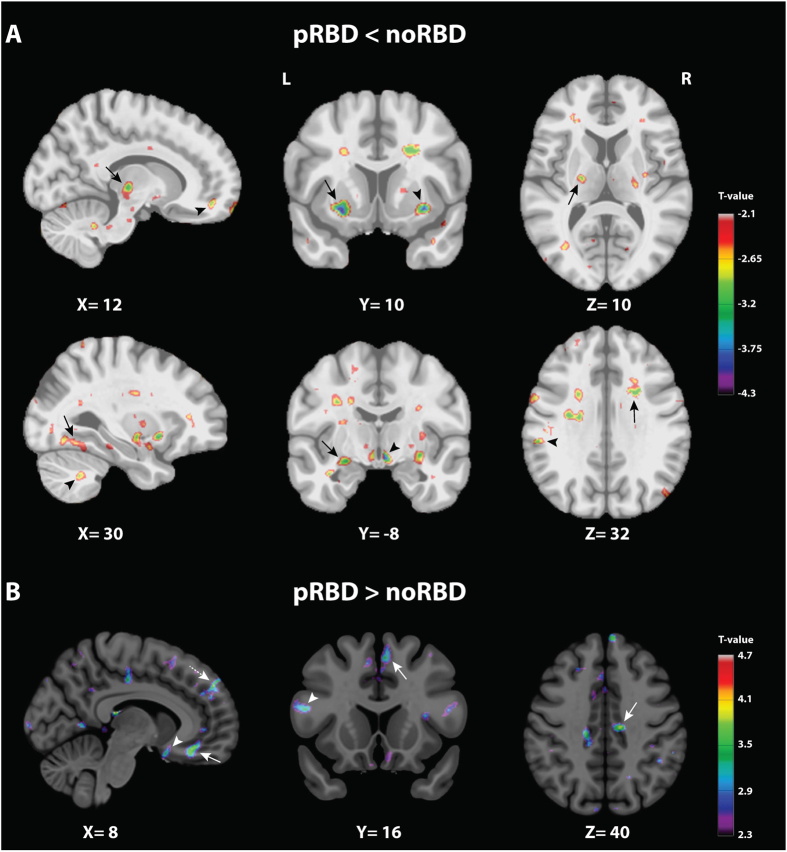
Volume change comparison between PD patients with (pRBD) and without (noRBD) probable RBD in sub-cortical and cortical areas. Thresholded *t* maps (at *P* < 0.05 corrected for multiple comparisons) superimposed onto Montreal Neurological Institute 152 brain imaging template. The color-coded bar represents t values and the display is thresholded at *P* < 0.05. (**A**) Areas of significantly smaller volume in pRBD. Upper level: Left, sagittal plane showing smaller volumes in the thalamus (arrow) and the anterior cingulate (arrow head). Middle, coronal plane showing smaller volumes in the putamen (arrow) and the claustrum (arrow head). Right, axial plane showing smaller volume in the posterior limb of the internal capsule (arrow). Lower level: Left, sagittal plane showing smaller volumes in the lingual gyrus (arrow) and the cerebellum (arrow head). Middle, coronal plane showing smaller volumes in the amygdala (arrow) and the hypothalamus (arrow head). Right, axial plane showing smaller volumes in the middle and anterior part of the superior longitudinal fasciculus (arrow), and the supramarginal gyrus (arrow head). (**B**) Areas of significantly larger volume in pRBD. Left, sagittal plane showing larger volume in an area encompassing the rectal gyrus and the orbitofrontal gyrus (arrow), the olfactory trigone (arrow head), and the medial prefrontal cortex (dashed arrow). Middle, coronal plane showing larger volume in the superior frontal gyrus (arrow) and the inferior frontal gyrus (arrow head). Right, axial plane showing larger volume in the mid-cingulate gyrus (arrow).

To evidence abnormalities associated with the probable presence of RBD in PD, a DBM comparison between the two PD groups was made (Table 2). At the level of the brainstem (Fig. 1), a smaller volume was observed in pRBD compared to noRBD in a region encompassing the PMT, including the oral pontine RF, the pedunculopontine tegmental nucleus (PPT), the laterodorsal and sublaterodorsal tegmental nuclei (LDT, SubLDT) and the ventral periaqueductal gray matter (PAG) along with the raphe nuclei. The region of smaller volume extended rostrally into the dorsal mesencephalic tegmentum including areas compatible with the mesencephalic RF and the PAG. It also extended caudally to affect areas compatible with the caudal pontine RF, the locus coeruleus/subcoeruleus, and the base of the pons. At the level of the medulla, smaller volume mainly affected the region of the medullary RF. In the forebrain (Fig. 2), a significantly smaller volume was observed in pRBD in the hypothalamus, thalamus, putamen, and amygdala. At the cortical level, smaller grey matter volumes included the anterior cingulate cortex. Besides these smaller volumes, patients with pRBD showed a larger volume of grey matter in several regions, including the olfactory trigone, medial prefrontal cortex, superior and inferior frontal gyri (Fig. 2, Table 2).

When focusing the analysis on the PMT area only, the pRBD showed a significantly smaller relative PMT volume compared to noRBD (p = 0.003) (Supp. Fig. 1B).

**Figure 3:**
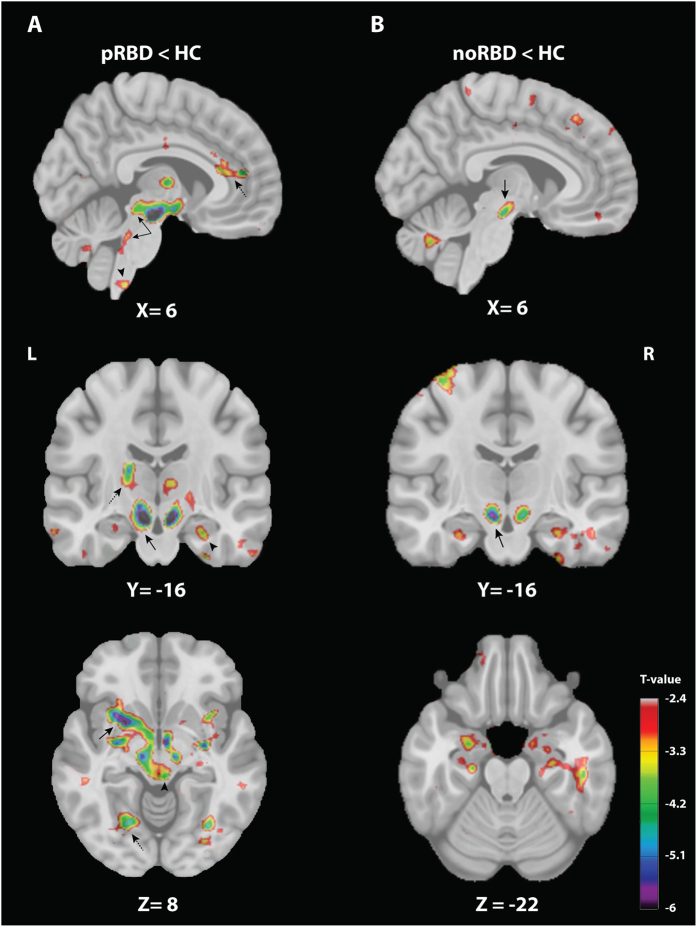
Volume change comparison between PD patients with (pRBD) and without (noRBD) probable RBD and healthy controls (HC). Thresholded *t* maps (at *P* < 0.05 corrected for multiple comparisons) superimposed onto Montreal Neurological Institute 152 brain imaging template. The color-coded bar represents t values and the display is thresholded at *P* < 0.05. (**A**) DBM group comparison between pRBD and HC showing areas of significantly smaller volume in pRBD in sub-cortical and cortical regions. Top, sagittal plane showing smaller volumes in the PMT (arrow), the ventral medulla (arrow head) and the anterior cingulate (dashed arrow). Middle, coronal plane showing smaller volumes in the substantia nigra (arrow), the hippocampus (arrow head) and the thalamus (dashed arrow). Bottom, axial plane showing smaller volumes in the putamen (arrow), the hypothalamus (arrow head) and the lingual gyrus (dashed arrow). (**B**) DBM group comparison between noRBD and HC showing areas of significantly smaller volume in noRBD, encompassing the substantia nigra (arrows).

To further evaluate whether these structural modifications were more specifically linked to the presence of RBD rather than PD itself, neuroimaging data of the two PD groups were respectively compared to HC (Fig. 3, Supp. Table 1). As expected, the vast majority of structural modifications associated with pRBD compared to noRBD were also observed in pRBD compared to HC, with the notable exception of the lower volume in the substantia nigra. The latter was found in both pRBD compared to HC and noRBD compared to HC (Fig. 3), but not in the comparison between the two PD groups (Figs1 and 2).

The correlation analyses did not reveal any significant association between relative volume differences in the predefined regions of interest (see methods section) and their corresponding clinical characteristics.

Finally, a smaller subsample of controls without PD met the RBDSQ criteria for probable idiopathic RBD (piRBD, n = 17) and were thus considered in the supplementary analyses where they were compared to a matched subpopulation of HC (n = 17, see supplementary data). This analysis showed several volume differences, including a lower volume in the PMT with piRBD (Supp. Table 2, Supp. Fig. 2).

## Discussion

Using DBM on a large sample of PD patients, we demonstrated extensive subcortical and cortical structural abnormalities associated with RBD in PD, whereas two previous neuroimaging studies on PD patients with RBD demonstrated only subtle (pontine) structural alterations but no statistically significant difference in regional brain volumes^19^,^23^. At the subcortical level, we found that pRBD patients manifest distinctly smaller volumes in the brainstem (PMT and medullary RF), cerebellum, diencephalon (hypothalamus and thalamus), striatum (putamen), and limbic system (amygdala). At the cortical level, they manifest distinct patterns of both smaller (anterior cingulate) and larger (olfactory) grey matter volumes. No significant volume difference was observed in the substantia nigra between the two PD groups, whereas this structure showed significantly smaller volume in pRBD and noRBD compared to HC, indicating that changes in the substantia nigra were specifically linked to PD.

At the level of the brainstem, smaller volumes in pRBD were mainly localized in a large cluster within the PMT in PD patients. A smaller volume in a more restricted region of the PMT was also found when comparing piRBD and HC. A few published case reports have described the occurrence of RBD symptoms with lesions encompassing the pontine tegmentum^2^,^24^, which offered early indications that alterations in this region might be involved in the loss of REM muscle atonia. Neuroimaging studies of idiopathic RBD evaluated the presence of structural alterations of this region in the absence of macroscopic lesions^9^,^10^,^11^,^12^. A relative lack of consistency in the results precluded a firm conclusion since only one of these studies demonstrated smaller volume in the pontine tegmentum^11^, two others showed only subtle structural changes in this region on MRI diffusion sequences^9^,^10^, and another one reported no volume change in this area^12^. In addition, previous neuroimaging studies of RBD in the presence of PD did not demonstrate any significant volume change of the PMT^19^,^23^,^25^. The present finding on 69 PD patients with probable RBD thus constitutes the first evidence for a significant PMT volume loss in PD with RBD, and thus demonstrates the key importance of PMT alteration in the loss of REM atonia underlying RBD. Further caudally in the brainstem, volume decrease also involved the medullary RF, which is known to be part of the neuronal system promoting muscle atonia during REM sleep^3^,^26^. Animal studies and a human case report have shown that lesions in the medullary RF are associated with loss of REM muscle atonia and RBD^5^,^27^. Collectively, the changes observed through the brainstem indicate that RBD involves degeneration of broadly distributed neural populations. These could include cholinergic neurons of the PMT (PPT, LDT and SubLDT), which have been shown to promote REM sleep with muscle atonia^7^,^28^, and for which evidence of loss has been reported in imaging studies of PD with RBD^29^. More numerous glutamatergic neurons are also present in this region and implicated in the control of muscle atonia^3^,^28^,^30^. GABAergic neurons are also present in the PMT and include neurons that are maximally active during REM sleep and can inhibit other neurons, including noradrenergic neurons of the locus coeruleus and serotonergic neurons of the raphe, which promote arousal with muscle tone^26^,^28^. Here, a smaller volume was found in pRBD in regions encompassing the locus coeruleus/subcoeruleus and raphe, in line with a previous MRI study^19^. Such results indicate that there are distributed neuroanatomical/chemical alterations affecting multiple cell populations to differing degrees, some of which normally play opposing roles in the occurrence of REM sleep and muscle atonia. The symptoms of RBD thus likely result from imbalances due to alterations in these diverse sleep-wake executive and regulatory neurochemical systems^5^,^7^,^28^. Although these observed changes in brain volume might in theory reflect losses in both neuronal and non-neuronal tissues (e.g., glial cells), evidences of neuronal degeneration (e.g., cholinergic, dopaminergic) were previously shown in PD with RBD and idiopathic RBD patients^29^,^31^.

In the forebrain, reduced volume with pRBD was observed in the thalamus – in agreement with a previous MRI study^25^ – as well as in the hypothalamus, which might be partly responsible for the increased sleepiness levels of these patients^32^. Moreover, orexin neurons, which play a critical role in maintenance of arousal and muscle tone^33^, are located in the hypothalamic region where volume reduction was seen here. Loss of orexin neurons, which has been shown to occur with PD^34^, could thus be associated with increased sleepiness in RBD patients, but also with the reported appearance in some RBD patients of narcolepsy with cataplexy^35^. Other volume decreases affected structures involved in motor control, such as the cerebellum and putamen, in line with previous MRI studies in idiopathic RBD^11^,^36^. Smaller volumes in these regions may reflect the decreased striatal dopaminergic innervation^31^, as well as the more severe motor impairments^37^ observed in patients with RBD. The smaller volumes in the limbic system (amygdala, anterior cingulate) might contribute to the changes in mood scores observed in pRBD, in line with the greater depression levels previously reported in this population^15^. At the cortical level, pRBD showed larger grey matter volumes including areas that belong and surround the olfactory cortex such as the olfactory trigone, the gyrus rectus and the orbitofrontal cortex. Previous MRI studies also showed structural changes in the olfactory cortex of idiopathic RBD patients^10^,^12^. Olfactory deficits have been reported in idiopathic RBD patients^38^ as well as in PD patients^39^. In the present dataset, olfactory deficits were found more pronounced in pRBD compared to the other groups. Therefore, the larger volume reported might represent a compensatory phenomenon to the more severe clinical olfactory deficits observed in pRBD.

Finally, since polysomnography data were not available in the PPMI dataset at the time of this analysis, they could not be used to confirm the definitive diagnosis of RBD. While this is a limitation of the current study, RBDSQ scores used to identify RBD were shown to have high sensitivity (96%) and specificity (85%)^20^ and were validated across several studies and population samples, including PD patients^40^,^41^,^42^,^43^. In addition, validated RBD questionnaires have been increasingly used in recent studies, in which RBDSQ-based probable RBD was associated with other clinical markers of neurodegeneration, illustrating the relevance of such questionnaires in studies with large sample sizes^44^.

In the present study, no significant correlation was observed between volume changes in specific regions of interest and selected clinical parameters, such as RBDSQ, self-reported mood scales or cognitive measures. This suggests that clinical differences associated with RBD in PD might not result from the impairment of specific brain structures considered separately, but rather from the alteration and resulting imbalance in complex systems and neural networks regulating sleep-wake cycles, sensorimotor control, as well emotional and cognitive processes.

## Conclusions

Resorting to brain morphometric analyses with DBM in a large cohort of PD patients, the present study revealed extensive structural abnormalities associated with RBD. These changes prominently involved volume decreases in the PMT, providing strong support for a consistent loss of neurons in this region with RBD, and thus emphasizing the key role of PMT in the control of muscle atonia during human REM sleep. Beyond the PMT, our results show for the first time the presence of distributed cortical and subcortical structural modifications associated with the presence of RBD in PD. This complex collection of neuroanatomical changes might contribute to the altered regulation of sleep-wake states and motor activity underlying RBD, as well as to the more severe clinical deficits in non-motor domains (e.g., olfaction, sleepiness, cognition) that characterize PD patients with RBD.

## Materials and Methods

### Subjects

All MRI and clinical data were extracted from the PPMI database: http://www.ppmi-info.org/ 22. The data from this database are openly accessible through a standard application process. The PPMI program was approved by the Institutional Review Board of each participating site. All participants to the PPMI gave their written informed consent to participate to the program. In accordance with PPMI policies, our manuscript was reviewed by the PPMI Data and Publications Committee for administrative approval. The analyses presented in this article were thus carried out in accordance with the approved PPMI guidelines.

PD patients in the PPMI cohort were diagnosed within the last two years, and were all drug naïve. Given the absence of polysomnography in the current PPMI database, the probable presence or absence of RBD in PD patients was based on RBDSQ. This 10-item structured questionnaire, which centres on the characteristics of dreams and dream enactment behaviours, has been validated as a screening tool for RBD^20^,^40^,^41^,^42^,^43^. A RBDSQ score ≥5 demonstrated a sensitivity of 96% and specificity of 56% for RBD detection^20^. In addition, single-item analysis revealed that each of three specific questions from RBDSQ (items 5, 6.3 and 6.4) had specificity above 85% for RBD diagnosis. Here, to ensure optimal sensitivity (96%) and specificity (>85%) of classification, PD patients combining both RBDSQ score ≥5 and positive response to item 5, 6.3 or 6.4 were considered as probable RBD (pRBD; n = 69). PD patients presenting a RBD score <5 were considered as without RBD (noRBD; n = 240). We extracted the data of subjects who completed the MRI scanning and the RBDSQ. For subjects with repeated RBDSQ assessments, we selected the RBDSQ that was the closest to the MRI scanning day. Control subjects without PD (i.e., had no history of PD in their first-degree blood relative) were also available in the PPMI database and were added to the comparison. Using the same RBDSQ criteria mentioned above, controls were classified as without RBD (healthy controls, HC) or with probable RBD (probable idiopathic RBD, piRBD). The main analysis aimed at comparing the two groups of PD patients (pRBD and noRBD), to identify structural abnormalities associated with probable RBD. To further differentiate structural abnormalities associated with RBD from those related to PD, secondary analyses were made to compare pRBD and noRBD groups to HC (n = 138) respectively. Finally, supplementary analyses compared piRBD and HC (Supp. Table 2 and Fig. 2)

### Clinical characteristics

We compared clinical scores between pRBD, noRBD and HC to provide insight into the significance of structural brain differences. To evaluate the severity of PD symptoms, (MDS-UPDRS) I, II and III as well as Modified Schwab and SE-ADL were used. Neuropsychiatric scales included the ESS, Geriatric Depression Scale (GDS), State-Trait Anxiety Inventory (STAI) and UPSIT. Cognitive assessments included the Montreal Cognitive Assessment (MoCA), SVFT, SDMT and BJLO. Scales for Outcomes in PD-Autonomic (SCOPA-AUT) was used to evaluate autonomic dysfunctions. One-way analyses of variance followed by Bonferroni post-hoc comparisons (for continuous variables), and Chi-square tests (for categorical variables) were performed in SPSS (version 21) to reveal significant differences in these parameters between the three groups (p < 0.05; Table 1).

### MRI acquisition

Non-contrast enhanced 3D volumetric T1-weighted brain MRI scans were acquired using 1.5 or 3 Tesla scanners. A majority of subjects were scanned using a 3 Tesla MR scanner: out of 69 pRBD, 26 were scanned with 1.5 Tesla MR scanner and 43 with a 3 Tesla; out of 240 noRBD, 73 were scanned at 1.5 Tesla and 167 at 3 Tesla; out of 138 HC, 45 were scanned at 1.5 Tesla and 93 at 3 Tesla. There was no significant difference in the proportion of subjects scanned at 1.5 Tesla vs 3 Tesla across the three groups (Chi square test, p = 0.520). To minimize bias in data between sites, the PPMI core optimized the acquisition sequence across sites to maximize comparability of data in the study. Typical MRI parameters were as follows: repetition time 5–11 ms; echo time 2–6 ms; slice thickness 1–1.5 mm; inter slice gap 0 mm; voxel size 1*1*1.2 mm; matrix 256 * minimum 160. Details can be found at http://www.ppmi-info.org/wp-content/uploads/2010/07/Imaging-Manual.pdf.

### MRI analysis

The MINC software (*MINC Tool Kit, Montreal Neurological Institute, McGill University, Montreal, Canada*) was used for data analysis and images generation. Quality control of the images was manually verified for each step of processing. If necessary, data was manually initialized (e.g., registration). Pre-processing steps were applied prior to estimating the deformations: (1) N3 non-uniformity correction; (2) linear normalization of individual scan’s intensity range to (0–100) by a single linear histogram scaling; (3) automatic linear (nine parameters) registration to the ICBM 152 stereotaxic space; and (4) brain mask creation. Only voxels within the brain volume after linear mapping into stereotaxic space were used for nonlinear registration. Using nonlinear transformations, voxel-wise deformations were calculated to make inferences about regionally specific differences between populations. A robust automated processing pipeline was used to control the bias due to multisite MRI scanning (i.e., magnetic field strength) and has been successfully applied to a number of multi-site collaborative projects^45^,^46^,^47^,^48^,^49^. Moreover, to further account for these multisite-related differences, magnetic field strength was also controlled along with age and gender in all parametric statistical tests.

DBM was used to compare brain morphometric changes between groups^50^. In brief, the local volume difference at each voxel was computed and used to measure possible brain tissue growth or loss. To compare brain volumetric changes between groups, independent voxel-by-voxel t-tests were used and results were thresholded at P value <0.05 after correction for multiple comparisons using the false discovery rate (FDR). To avoid reporting insignificant volume changes, only clusters with ≥50 contiguous voxels were considered^51^,^52^.

Mean volume changes of the PMT area were additionally calculated and compared (independent t-test) between pRBD and noRBD (p < 0.05). The PMT was manually defined on the Montreal Neurological Institute 152 nonlinear 2009 ICBM template and the integral of the determinant of the Jacobian of the individual’s estimated deformation field was computed within this mask (Supp. Fig. 1A).

Finally, to assess whether differences in clinical characteristics between PD patients with and without RBD might be associated with regional brain volume changes, correlation analyses between volume changes and clinical variables were performed across the two patients groups using Pearson’s linear correlations (corrected for age, PD duration since diagnosis, and MRI scan parameters; p < 0.05). We restricted our correlations to the regions most consistently reported in neuroimaging studies of idiopathic RBD^9^,^12^,^36^,^53^. The following correlations were thus performed: the volume change in the PMT and the RBDSQ score; the olfactory trigone and the UPSIT; the putamen and the MDS-UPDRS III; the anterior cingulate cortex and the GDS; the medial prefrontal cortex and cognitive tests (MOCA, SDMT, SVF). These regions of interest were manually defined on the ICBM template and the relative volume estimated as described above. The level of significance was set at p < 0.05.

## Additional Information

**How to cite this article**: Boucetta, S. *et al.* Structural Brain Alterations Associated with Rapid Eye Movement Sleep Behavior Disorder in Parkinson’s Disease. *Sci. Rep.* **6**, 26782; doi: 10.1038/srep26782 (2016).

## Supplementary Material

Supplementary Information

## Figures and Tables

**Table 1:** Clinical characteristics.

Parameters	pRBD^a^(N = 69)	noRBD^b^(N = 240)	HC^c^(N = 138)
Demographics			
Age^#^	60.93 ± 9.16	61.62 ± 9.84	59.92 ± 10.83
Sex (M:F)^§^	52:17	149:91	90:48
Education (Years)^#^	15.86 ± 2.34	15.63 ± 2.90	16.28 ± 2.92
Parkinson’s Disease Duration since Diagnosis (Months)^†^	6.29 ± 6.62	6.85 ± 6.66	N/A
Family History of Parkinson’s Disease^§^	30.4%^c^	23.3%^c^	5.1%^a,b^
RBDSQ Score^#^	8.12 ± 1.97^b,c^	2.45 ± 1.15^a,c^	1.93 ± 1.33^a,b^
Parkinson’s disease rating scales			
UPDRS I^#^	7.91 ± 4.44^b,c^	4.88 ± 3.57^a,c^	2.76 ± 3.18^a,b^
UPDRS II^#^	8.41 ± 5.30^b,c^	5.38 ± 3.91^a,c^	0.38 ± 0.92^a,b^
UPDRS III^#^	22.45 ± 9.46^c^	21.14 ± 8.83^c^	1.03 ± 2.13^a,b^
SE-ADL^†^	91.96 ± 5.77^b^	93.92 ± 6.10^a^	N/A
Other clinical scales^#^:			
Epworth Sleepiness Scale	6.94 ± 4.66^b,c^	5.50 ± 3.29^a^	5.25 ± 3.46^a^
University of Pennsylvania Smell Identification Test	20.67 ± 8.67^b,c^	22.66 ± 8.14^a,c^	34.30 ± 4.00^a,b^
Geriatric Depression Scale	3.04 ± 2.45^b,c^	2.00 ± 2.24^a,c^	1.39 ± 2.41^a,b^
State-Trait Anxiety Inventory - State	38.19 ± 9.00^b,c^	34.50 ± 7.43^a,c^	31.82 ± 6.37^a,b^
State-Trait Anxiety Inventory - Trait	35.16 ± 10.19^b,c^	31.38 ± 9.12^a,c^	28.72 ± 7.79^a,b^
Montreal Cognitive Assessment	27.77 ± 2.63	28.16 ± 2.36	28.20 ± 1.11
Symbol Digit Modalities Test	40.04 ± 10.28^c^	42.22 ± 9.71^c^	46.80 ± 10.18^a,b^
Semantic Verbal Fluency Test (Scaled Score)	10.19 ± 2.78^b,c^	11.05 ± 2.97^a^	11.19 ± 2.88^a^
Visuospatial skills (Benton’s Judgment of Line Orientation)	12.22 ± 2.34^b,c^	12.94 ± 1.94^a^	13.11 ± 2.10^a^
SCOPA-Autonomic	12.30 ± 6.51^b,c^	8.22 ± 5.28^a,c^	5.33 ± 3.66^a,b^

Means ± standard deviations are presented and statistically compared. Statistical differences, with significance at P < 0.05, are indicated between groups by corresponding letters; ^a^Parkinson’s disease patients with probable RBD, ^b^Parkinson’s disease patients without RBD and ^c^healthy controls.

^#^Based on *Post-hoc* Bonferroni pairwise. ^†^Based on Student *t* test. ^§^Based on Pearson’s *χ*^2^ test.

Abbreviations: F: Female, M: Male, N: Number of subjects, RBDSQ: Rapid eye movement sleep behaviour disorder screening questionnaire, SCOPA: Scales for outcomes in Parkinson’s disease, SE-ADL: Modified Schwab and England activities of daily living scale, UPDRS: Unified Parkinson’s disease rating scale.

**Table 2:** Areas of significant volume difference in PD patients with probable RBD (pRBD) compared to PD patients without (noRBD).

Area	X^a^	Y^a^	Z^a^	T-score^b^	P-value^c^
pRBD < noRBD					
Pontomesencephalic Tegmentum					
Left	−4	−28	−22	−3.70	0.00011
Right	4	−30	−20	−2.72	0.00312
Base of the Pons					
Left	−5	−18	−36	−3.50	0.00029
Right	4	−18	−34	−2.72	0.00291
Medullary Reticular Formation					
Left	−4	−42	−62	−2.71	0.00364
Cerebellum – Deep Nuclei					
Left	−24	−46	−34	−3.60	0.00022
Right	24	−46	−34	−3.90	0.00006
Cerebral Peduncle					
Left	−12	−18	−20	−2.72	0.00376
Right	12	−16	−18	−2.33	0.01107
Hypothalamus					
Left	−4	−4	−10	−3.31	0.00056
Right	4	−6	−10	−4.29	0.00001
Thalamus					
Left	−19	−18	16	−3.31	0.00059
Right	12	−20	0	−3.50	0.00025
Putamen					
Left	−24	10	−10	−4.19	0.00002
Right	32	−6	−6	−3.31	0.00062
Claustrum					
Left	−29	12	−8	−3.50	0.00025
Right	32	10	−8	−3.99	0.00004
Internal Capsule – Posterior lobe					
Left	−22	−12	10	−3.11	0.00125
Right	24	−18	10	−2.91	0.00210
Amygdala					
Left	−26	−8	−12	−3.50	0.00023
Right	30	−8	−8	−3.21	0.00069
Lingual Gyrus					
Left	−22	−68	−15	−3.21	0.00084
Right	32	−70	−10	−2.91	0.00150
Supramarginal Gyrus					
Left	−56	−32	32	−3.21	0.00090
Anterior cingulate					
Right	14	50	−12	−3.21	0.00077
Superior Longitudinal Fasciculus					
Left	−22	−12	32	−3.51	0.00025
Right	22	8	32	−3.50	0.00034
Inferior Longitudinal Fasciculus					
Left	−36	−4	−24	−3.99	0.00004
Right	34	2	−20	−3.90	0.00006
**pRBD** > **noRBD**					
Olfactory Trigone					
Left	−6	18	−23	2.58	0.00451
Right	6	12	−18	3.66	0.00019
Rectal Gyrus/Orbitofrontal Gyrus					
Right	10	36	−14	3.96	0.00003
Medial Prefrontal Cortex					
Left	−4	54	36	3.27	0.00064
Right	6	54	38	4.25	0.00001
Superior Frontal Gyrus					
Left	−6	14	50	2.98	0.00165
Right	5	20	58	3.86	0.00008
Inferior Frontal Gyrus					
Left	−51	16	16	3.37	0.00039
Right	56	26	16	3.76	0.00009
Mid-Cingulate Gyrus					
Left	−16	−24	40	3.76	0.00009
Right	12	−18	40	4.06	0.00003
Superior Temporal Gyrus					
Left	−64	−48	20	4.16	0.00002
Right	69	−42	16	4.65	0.00000

^a^Based on Montreal Neurological Institute 152 brain imaging template; ^b^Peak voxel T-score; ^c^Peak voxel P-value.
