# Supplementary material for: Structural Brain Alterations Associated with Rapid Eye Movement Sleep Behavior Disorder in Parkinson’s Disease
Source: Sci Rep. 2016 Jun 1;6:26782. doi: 10.1038/srep26782 (PMC4887790; doi:10.1038/srep26782)
Supplement: Supplementary Information [file srep26782-s1.docx]

**Structural Brain Alterations Associated with Rapid Eye Movement Sleep Behavior Disorder in Parkinson’s Disease**

*Running title: Neuroimaging of REM Sleep Behavior Disorder*

Soufiane Boucetta, PhD^1,3^, Ali Salimi, BSc^1,3^, Mahsa Dadar, MSc^2^, Barbara E Jones, PhD^2^, D. Louis Collins, PhD^2^, and Thien Thanh Dang-Vu, MD, PhD^1,3*^

*^1^ Center for Studies in Behavioural Neurobiology, PERFORM Center and Dpt of Exercise Science, Concordia University, 7141 Sherbrooke St. West, Montréal, Québec, Canada H4B 1R6*

*^2^ Montreal Neurological Institute, McGill University, 3801 University Street, Montréal, Québec, Canada H3A 2B4*

*^3^Centre de Recherche de l’Institut Universitaire de Gériatrie de Montréal and Dpt of Neurosciences, Université de Montréal, 4545 Chemin Queen Mary, Montréal, Québec, Canada H3W 1W4*

^*^Corresponding author:

T. T. Dang-Vu, MD PhD

Center for Studies in Behavioral Neurobiology (CSBN), PERFORM Center

Dpt of Exercise Science

Concordia University

7141 Sherbrooke St. West, SP 165.27

*Montréal* H4B 1R6

Tel: (514) 848-2424, Ext 3364

Institut Universitaire de Gériatrie de Montréal (IUGM)

Dpt of Neurosciences

Université de Montréal

4545 chemin Queen-Mary, M6822

*Montréal* H3W 1W4

Tel: (514) 340-3540, Ext 3991

Email: tt.dangvu@concordia.ca

| **Supplementary Table 1:** **Areas of significant volume difference in PD patients (pRBD and noRBD) compared to** **healthy controls** | | | | | | | | | | | | |
| --- | --- | --- | --- | --- | --- | --- | --- | --- | --- | --- | --- | --- |
|  | | **pRBD < HC** | | | | | **noRBD < HC** | | | | |  |
| **Area** | **X** ^a^ | | **Y** ^a^ | **Z** ^a^ | **T-score** ^b^ | **P-value** ^c^ | **X** ^a^ | **Y** ^a^ | **Z** ^a^ | **T-score** ^b^ | **P-value** ^c^ |  |
| Pontomesencephalic Tegmentum |  | |  |  |  |  |  |  |  |  |  |  |
| Left | -2 | | -34 | -11 | -3.12 | 0.00113 | - | - | - | - | - |  |
| Right | 4 | | -32 | -10 | -3.25 | 0.00070 | - | - | - | - | - |  |
| Medullary Reticular Formation |  | |  |  |  |  |  |  |  |  |  |  |
| Left | -4 | | -38 | -60 | -3.03 | 0.00137 | - | - | - | - | - |  |
| Cerebellum – Deep Nuclei |  | |  |  |  |  |  |  |  |  |  |  |
| Left | -22 | | -40 | -34 | -3.48 | 0.00028 | - | - | - | - | - |  |
| Right | 24 | | -46 | -34 | -3.90 | 0.00026 | 6 | -68 | -35 | -3.64 | 0.00012 |  |
| Substantia Nigra |  | |  |  |  |  |  |  |  |  |  |  |
| Left | -6 | | -20 | -16 | -5.88 | 0.00000 | -8 | -16 | -14 | -6.00 | 0.00000 |  |
| Right | 6 | | -20 | -16 | -5.06 | 0.00000 | 10 | -16 | -12 | -4.77 | 0.00000 |  |
| Hypothalamus |  | |  |  |  |  |  |  |  |  |  |  |
| Left | -6 | | -4 | -10 | -3.43 | 0.00036 | - | - | - | - | - |  |
| Right | 6 | | -8 | -10 | -4.43 | 0.00008 | - | - | - | - | - |  |
| Thalamus |  | |  |  |  |  |  |  |  |  |  |  |
| Left | -18 | | -16 | 14 | -3.57 | 0.00026 | - | - | - | - | - |  |
| Right | 16 | | -30 | 4 | -3.43 | 0.00036 | - | - | - | - | - |  |
| Putamen |  | |  |  |  |  |  |  |  |  |  |  |
| Left | -22 | | 8 | -11 | -4.38 | 0.00008 | - | - | - | - | - |  |
| Right | 32 | | -2 | 3 | -3.61 | 0.00019 | - | - | - | - | - |  |
| Claustrum |  | |  |  |  |  |  |  |  |  |  |  |
| Left | -29 | | 12 | -8 | -3.71 | 0.00013 | - | - | - | - | - |  |
| Right | 32 | | 10 | -8 | -2.94 | 0.00187 | - | - | - | - | - |  |
| Amygdala |  | |  |  |  |  |  |  |  |  |  |  |
| Left | -24 | | -8 | -12 | -4.52 | 0.00008 | - | - | - | - | - |  |
| Right | 30 | | 2 | -16 | -4.38 | 0.00008 | - | - | - | - | - |  |
| Hippocampus |  | |  |  |  |  |  |  |  |  |  |  |
| Left | -30 | | -10 | -26 | -3.30 | 0.00064 | -22 | -20 | -20 | -4.11 | 0.00003 |  |
| Right | 28 | | -16 | -22 | -2.67 | 0.00341 | 30 | -16 | -24 | -3.45 | 0.00028 |  |
| Lingual Gyrus |  | |  |  |  |  |  |  |  |  |  |  |
| Left | -19 | | -66 | -6 | -3.43 | 0.00037 | - | - | - | - | - |  |
| Right | 34 | | -68 | -6 | -3.43 | 0.00037 | - | - | - | -*- | - |  |
| Fusiform Gyrus |  | |  |  |  |  |  |  |  |  |  |  |
| Left | - | | - | - | - | - | -46 | -32 | -22 | -2.50 | 0.00541 |  |
| Right | 52 | | -22 | -26 | -3.21 | 0.00082 | 50 | -24 | -24 | -5.25 | 0.00000 |  |
| Supramarginal Gyrus |  | |  |  |  |  |  |  |  |  |  |  |
| Left | -56 | | -30 | 32 | -3.21 | 0.00082 | - | - | - | - | - |  |
| Orbitofrontal Gyrus |  | |  |  |  |  |  |  |  |  |  |  |
| Left | 16 | | 50 | -12 | -3.07 | 0.00117 | - | - | - | - | - |  |
| Right | 36 | | 0 | -28 | -3.80 | 0.00010 | - | - | - | - | - |  |
| Pre-Central Gyrus |  | |  |  |  |  |  |  |  |  |  |  |
| Left | - | | - | - | - | - | -40 | -24 | 58 | -4.39 | 0.00001 |  |
| Anterior cingulate |  | |  |  |  |  |  |  |  |  |  |  |
| Left | -6 | | 46 | 14 | -3.39 | 0.00040 | - | - | - | - | - |  |
|  | | **pRBD > HC** | | | | | **noRBD > HC** | | | | |  |
| Pontomesencephalic Tegmentum |  | |  |  |  |  |  |  |  |  |  |  |
| Left | - | | - | - | - | - | -12 | -26 | -24 | 3.74 | 0.00011 |  |
| Right | - | | - | - | - | - | 4 | -32 | -20 | 2.89 | 0.00165 |  |
| Base of the pons |  | |  |  |  |  |  |  |  |  |  |  |
| Left | - | | - | - | - | - | -8 | -14 | -26 | 3.36 | 0.00048 |  |
| Right | - | | - | - | - | - | 6 | -26 | -44 | 3.55 | 0.00021 |  |
| Medullary Reticular Formation |  | |  |  |  |  |  |  |  |  |  |  |
| Right | - | | - | - | - | - | 6 | -40 | -62 | 2.89 | 0.00206 |  |
| Olfactory Trigone |  | |  |  |  |  |  |  |  |  |  |  |
| Left | -8 | | 20 | -24 | 3.44 | 0.00039 | -16 | 18 | -24 | 3.17 | 0.00093 |  |
| Right | 6 | | 14 | -20 | 4.03 | 0.00008 | - | - | - | - | - |  |
| Gyrus Rectus |  | |  |  |  |  |  |  |  |  |  |  |
| Right | 8 | | 34 | -16 | 4.57 | 0.00005 | - | - | - | - | - |  |
| Medial Prefrontal Cortex |  | |  |  |  |  |  |  |  |  |  |  |
| Left | -2 | | 60 | 18 | 3.26 | 0.00066 | - | - | - | - | - |  |
| Right | 2 | | 50 | 28 | 2.81 | 0.00276 | - | - | - | - | - |  |
| Superior Frontal Gyrus |  | |  |  |  |  |  |  |  |  |  |  |
| Right | 6 | | 20 | 58 | 2.99 | 0.00171 | - | - | - | - | - |  |
| Inferior Frontal Gyrus |  | |  |  |  |  |  |  |  |  |  |  |
| Left | -46 | | 4 | 19 | 3.40 | 0.00045 | - | - | - | - | - |  |
| Right | 56 | | 23 | 20 | 3.04 | 0.00150 | - | - | - | - | - |  |
| Mid-Cingulate Gyrus |  | |  |  |  |  |  |  |  |  |  |  |
| Left | -2 | | 6 | 40 | 3.13 | 0.00108 | - | - | - | - | - |  |
| Right | 8 | | -20 | 40 | 2.99 | 0.00179 | - | - | - | - | - |  |
| Posterior Cingulate Gyrus |  | |  |  |  |  |  |  |  |  |  |  |
| Left | - | | - | - | - | - | -8 | -34 | 30 | 3.27 | 0.00052 |  |

^a^ Based on Montreal Neurological Institute 152 brain imaging template; ^b^ Peak voxel T-score; ^c^ Peak voxel P-value.

| **Supplementary Table 2: Areas of significant volume difference in piRBD compared to** **healthy controls** | | | | | |
| --- | --- | --- | --- | --- | --- |
| **Area** | **X** a | **Y** a | **Z** a | **T-score** b | **P-value** c |
| **piRBD < HC** | | | | | |
| Pontomesencephalic Tegmentum | | | | | |
| Right | 6 | -33 | -14 | -2.37 | 0.01311 |
| Putamen | | | | | |
| Left | -18 | 7 | -8 | -2.25 | 0.01290 |
| Right | 22 | 6 | -2 | -2.62 | 0.00511 |
| Hippocampus | | | | | |
| Right | 25 | -14 | -20 | -2.28 | 0.01221 |
| Lingual Gyrus | | | | | |
| Right | 28 | -83 | -18 | -3.88 | 0.00016 |
| Fusiform Gyrus | | | | | |
| Left | -33 | -39 | -22 | -2.78 | 0.00318 |
| Precentral Gyrus | | | | | |
| Right | 42 | 0 | 50 | -3.22 | 0.00084 |
| Supramarginal Gyrus | | | | | |
| Right | 52 | -42 | 27 | -3.16 | 0.00101 |
| Anterior Cingulate | | | | | |
| Right | 9 | 48 | 11 | -2.38 | 0.00944 |
| Gyrus Rectus | | | | | |
| Right | 11 | 32 | -21 | -2.80 | 0.00307 |
| **piRBD > HC** | | | | | |
| Base of the Pons | | | | | |
| Right | 5 | -26 | -42 | 2.83 | 0.00243 |
| Dorsal Medulla | | | | | |
| Left | -6 | -43 | -48 | 3.80 | 0.00018 |
| Superior Parietal Gyrus | | | | | |
| Left | -40 | 0 | 50 | 5.13 | 0.00000 |
| Mid-Cingulate | | | | | |
| Left | -3 | 9 | 33 | 3.00 | 0.00129 |

a Based on Montreal Neurological Institute 152 brain imaging template; b Peak voxel T-score; c Peak voxel P-value

**
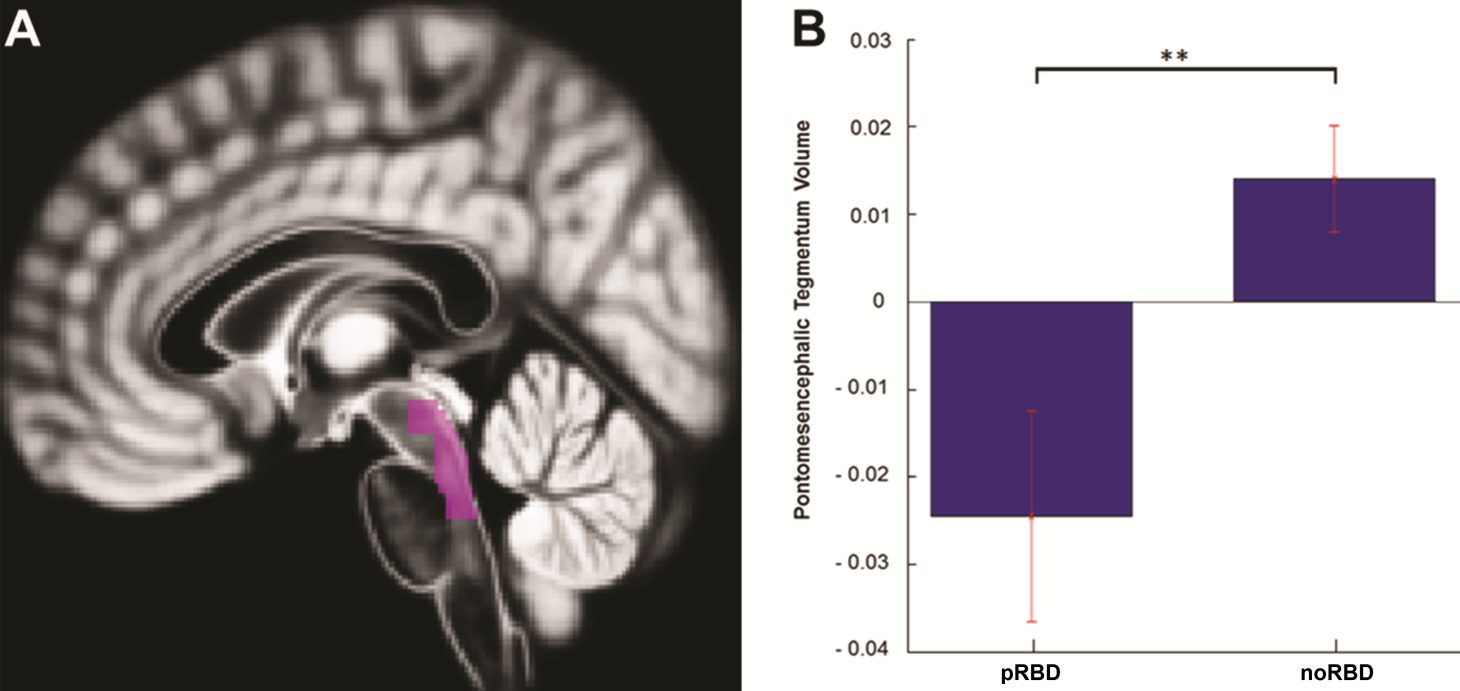
**

**Supplementary Figure 1: Volume comparison between PD groups (pRBD and noRBD) in the pontomesencephalic tegmentum (PMT) region.** (A) Definition of the region of interest corresponding to the PMT, using the Montreal Neurological Institute 152 nonlinear 2009 ICBM template. Sagittal section showing the delineation of the pontine tegmentum and dorsal mesencephalic tegmentum (purple), which include the caudal and oral pontine reticular formation, locus coeruleus and subcoeruleus nuclei, pedunculopontine, laterodorsal and sublaterodorsal tegmental nuclei, mesencephalic reticular formation, ventral periaqueductal gray and raphe nuclei. (B) PMT volume deformation (means ± SEM) were statistically compared (independent *t-test*) between pRBD (-0.025 ± 0.0120) and noRBD (0.014 ± 0.006). Volume deformation in arbitrary units. ** significance at p < 0.01.


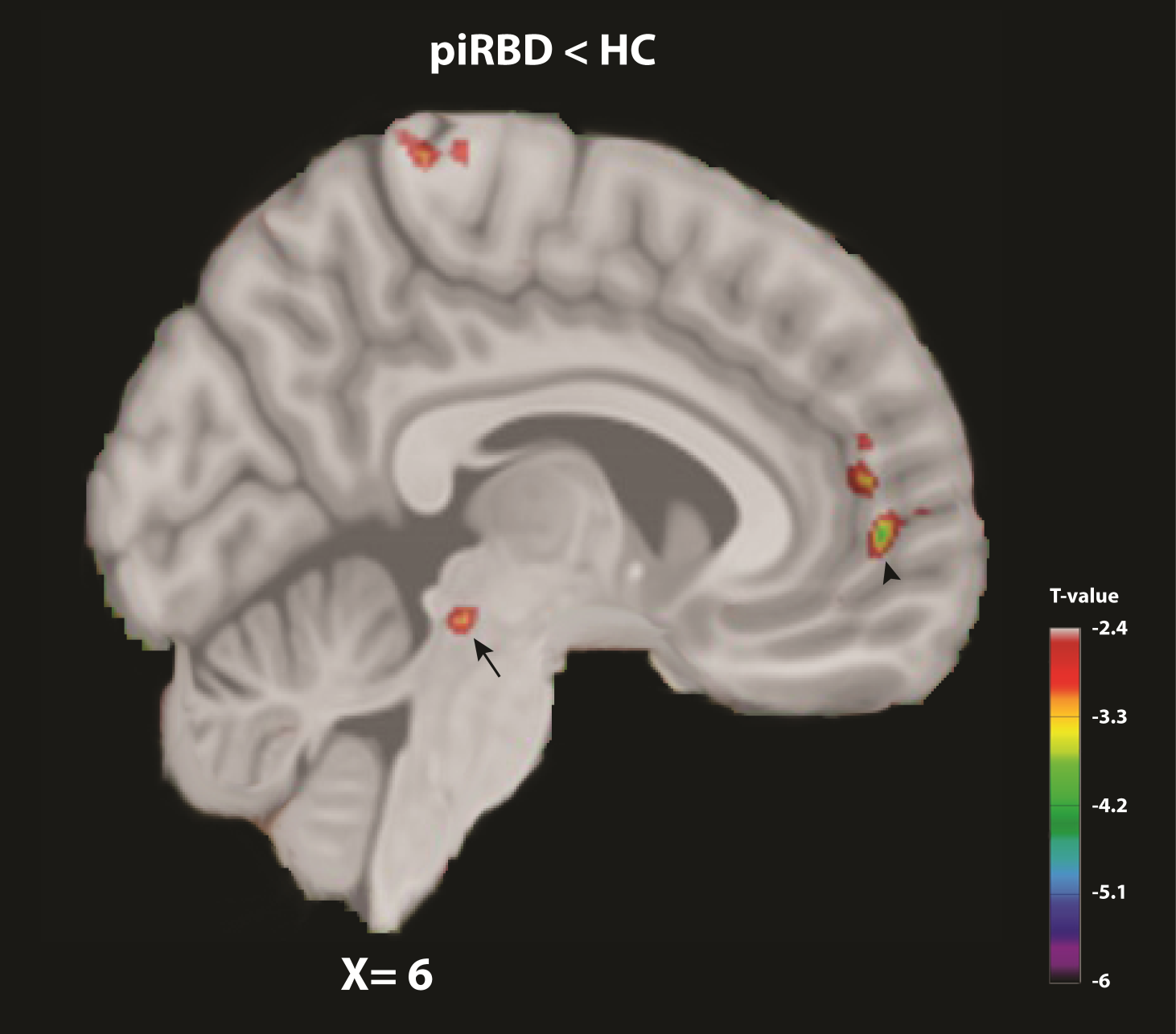


**Supplementary Figure 2: Volume change comparison between piRBD and healthy controls.** Thresholded t maps (at P < 0.05 corrected for multiple comparisons) superimposed onto Montreal Neurological Institute 152 brain imaging template. The color-coded bar represents t values and the display is thresholded at P < 0.05. Sagittal plane showing smaller volumes in the PMT (arrow) and anterior cingulate (arrow head).
